# Two Decades of Enhancing Children’s Environmental Health Protection at the U.S. Environmental Protection Agency

**DOI:** 10.1289/EHP1040

**Published:** 2016-12-01

**Authors:** Michael Firestone, Martha Berger, Brenda Foos, Ruth Etzel

**Affiliations:** U.S. Environmental Protection Agency, Office of Children’s Health Protection Washington, DC, USA

## Abstract

This article provides an overview of public health efforts by the U.S. Environmental Protection Agency (EPA) during the past two decades to protect children’s health from environmental hazards. It highlights examples of concrete steps and accomplishments toward improving environmental protection and health outcomes achieved through public policy, rules and regulations, increased scientific understanding, and public health messaging. Additionally, examples of future challenges for better understanding and improving children’s environmental health are discussed.

## Introduction

Children do not react like adults when exposed to environmental chemicals because their organ systems and metabolic capabilities are not fully developed. Children eat, breathe, and drink more, relative to their body mass, than adults. Their exposures to environmental chemicals differ from that of adults due to child-specific behaviors, such as hand-to-mouth and object-to-mouth activities, crawling on the ground, and breastfeeding. Meanwhile, research shows that exposures to environmental chemicals in homes, schools, food, and common household products may be associated with chronic conditions such as asthma, diabetes, obesity, attention-deficit disorders, learning disabilities, and autism (OUP 2013).

The efforts of the U.S. Environmental Protection Agency (EPA) to enhance consideration of the potential for early-life susceptibility stem from the 1993 National Research Council’s “Pesticides in the Diets of Infants and Children” (NRC 1993) and the subsequent 1996 Food Quality Protection Act (FQPA 1996). Twenty years ago, this legislative change to the regulation of pesticides initiated an unprecedented drive by the U.S. EPA to explicitly consider the unique exposure pathways of children versus those of adults. This summer, President Obama signed the Frank R. Lautenberg Chemical Safety for the 21st Century Act (2016), which reforms the 1976 Toxic Substance Control Act (1976) by directing the U.S. EPA to make an affirmative finding on the safety of a new chemical or a significant new use of an existing chemical before it is allowed into the marketplace. These revisions, 40 years in the making, provide a fresh opportunity for the U.S. EPA to further protect children’s environmental health by requiring the agency to address unreasonable risks to those who may be the most susceptible including children and pregnant women.

## Discussion

### Early Drivers for Focusing on Children’s Environmental Health

In 1988, when the U.S. EPA attempted to ease restrictions on several pesticides that posed a *de minimis* cancer risk to humans, the Natural Resources Defense Council brought suit and prevailed. The court stated that only Congress could change the provision designed to protect the public from carcinogens in processed foods and animal feeds in the Food Additives Amendment of 1958, known as the Delaney Clause (Hoyle 1996). Also in 1988, the Congress responded to public concerns about pesticide safety by requesting that the National Academy of Sciences study the issue of vulnerability of children to pesticides residues in foods. The resulting 1993 report “Pesticides in the Diets of Infants and Children” (NRC 1993) made several recommendations for changes in pesticide regulations to protect children’s environmental health, including accounting for the unique exposures of children and improving risk assessment methods to estimate the magnitude of the effects of these exposures on infants and children. This NRC report was a principal driver in ultimately elevating awareness among national policy makers of children’s vulnerability to toxic hazards, thereby moving U.S. environmental policy toward enhanced protection of children’s health and catalyzing research investment (Landrigan 2016). As a compromise to address both the public’s concern with protecting children and industry’s concern with the inflexibility of the Delaney Clause, the U.S. Congress revoked the Delaney Clause (Appel 1995) and then enacted the FQPA. It was passed unanimously by Congress and then signed into law by former President Clinton in 1996.

The FQPA included a number of provisions to better protect children starting with the use of an additional 10-fold safety factor when setting or reassessing tolerances for pesticides in foods unless adequate data are available to support a different factor. Other provisions that could address potential risk included screening pesticides for endocrine disruption, assessing aggregate exposure from both food and residential uses, and accounting for cumulative exposure to pesticides that have common mechanisms of toxicity.

### Building Children’s Environmental Health Protection at the U.S. EPA

Recognizing the imperative to protect the health of children, in 1995 the U.S. EPA issued its Policy on Evaluating Health Risks to Children (U.S. EPA 1995) directing the agency to explicitly and consistently take into account environmental health risks to infants and children in all risk characterizations and public health standards set for the United States (U.S. EPA 1995). In 2013, the U.S. EPA reaffirmed this policy and stressed the importance of “…encourag(ing) much-needed research to provide child-specific data required to thoroughly evaluate the health risks children in all life stages face from pollution in our air, land and water” (U.S. EPA 2013c).

Expanding the scope of federal protections, from the U.S. EPA to the myriad of agencies with responsibility for children’s health, former President Clinton issued Executive Order 13045 entitled “Protection of Children from Environmental Health Risks and Safety Risks” (The President 1997). The Executive Order required all federal agencies to assign a high priority to addressing health and safety risks to children, improve coordination of research priorities on children’s health, and ensure that federal standards take into account special risks to children.

The Executive Order required the creation of the “President’s Task Force on Environmental Health Risks and Safety Risks to Children” (https://www.epa.gov/children/presidents-task-force-environmental-health-and-safety-risks-children) to help implement the order across the federal government. To date, the task force, co-chaired by the U.S. EPA and the Department of Health and Human Services (DHHS), has focused on issues including lead, racial and ethnic asthma disparities, healthy homes, chemical exposures, and climate change.

The U.S. EPA created its Office of Children’s Health Protection (OCHP) in 1997. Although the agency’s mission has always been to protect human health and the environment, C.M. Browner and subsequent agency leaders have recognized that protecting children from environmental hazards required explicit and dedicated resources. The office promotes the protection of children by leading and/or partnering with other parts of the agency to ensure that actions by the U.S. EPA take into account any heightened risks faced by children, to help identify research gaps, and to encourage and expand health outreach efforts.

### Raising Awareness of Children’s Environmental Health

***Reaching parents.*** The U.S. EPA’s outreach and education efforts are designed to gather together the most important suite of key issues and public health messages on a given topic and share it widely. Outreach materials on topics such as asthma prevention, secondhand smoke, and impacts of climate change on children are available at the agency’s “Protecting Children’s Environmental Health” web site (https://www.epa.gov/children).

***Reaching health care providers.*** In the field of pediatrics, concerns about environmental influences on disease were infrequently considered in the 1990s. A 1995 Institute of Medicine report identified the lack of curricula on topics in environmental medicine in the training of physicians (IOM 1995). In order to increase the knowledge of practicing medical and nursing providers, the U.S. EPA has supported the development of and promoted the use of numerous training courses, information tools, manuals, and educational opportunities such as *Recognition and Management of Pesticide Poisonings* now in its 6th edition (U.S. EPA 2016). In 1999, with funding from the U.S. EPA, the American Academy of Pediatrics (AAP) first published its *Handbook of Pediatric Environmental Health*—a seminal accomplishment for building an academic foundation for children’s environmental health—the 3rd edition was published in 2012 (AAP 2012). Additional outreach materials can be found online at the agency’s “Children’s Environmental Health: Online Resources for Healthcare Providers” (https://www.epa.gov/children/childrens-environmental-health-online-resources-healthcare-providers).

Spurred by the lack of environmental health training among pediatricians, the “Pediatric Environmental Health Specialty Units” (PEHSU; http://www.pehsu.net/)—a network of experts in reproductive and children’s environmental health—has been jointly funded by the U.S. EPA and the Agency for Toxic Substances and Disease Registry to provide medical advice, outreach, and training about prevention, diagnosis, treatment, and management of environmental health illnesses for children. From 1999 to 2014, PEHSU conducted approximately 8,000 consultations and educational activities, reaching more than 700,000 individuals (Woolf et al. 2016).

***Informing safer school environments.*** Across the U.S. EPA, numerous efforts to address air quality, drinking water, and chemical safety in school environments represent ongoing federal assistance to local school districts in protecting America’s 53 million school children who will spend their formative years in environments potentially affected by numerous environmental threats. This effort was amplified with the Energy Independence and Security Act of 2007 (2007) that mandated “School Siting Guidelines” (U.S. EPA 2011d). These voluntary guidelines are designed to inform and improve the school siting decision-making process. Much additional information and tools to help establish, maintain, or enhance a school environmental health program can be found at U.S. EPA’s “Healthy Schools, Healthy Kids” website (https://www.epa.gov/schools).

### Addressing Early Life-Stage Risks

***Exposure assessment.*** In the early 2000s, the U.S. EPA began a process to consider how behavioral and physiological changes associated with childhood life stages could impact exposures to environmental hazards. Although environmental laws have referred to children as a subpopulation, two important risk assessment guidance documents published by the U.S. EPA in the mid-2000s emphasized the importance of distinguishing between population groups that form a relatively fixed portion of the population (e.g., groups based on ethnicity) and life stages or age groups that are inclusive of the entire population. The term “life stage” refers to a distinguishable time frame in an individual’s life characterized by unique and relatively stable behavioral and/or physiological characteristics that are associated with development and growth. Thus, the U.S. EPA is evolving to view childhood as a sequence of life stages, including infancy and adolescence through adulthood, rather than considering children as a fixed subpopulation.

In 2005, the U.S. EPA’s “Guidance on Selecting Age Groups for Monitoring and Assessing Childhood Exposures to Environmental Contaminants” (U.S. EPA 2005a) was published. This report considers unique behaviors of children such as breastfeeding, crawling, and hand-to-mouth and object-to-mouth activities. The development of a standard set of age groupings (see Table 1) is critical for developing risk assessments that adequately consider early life exposures and for focusing future research and data collection efforts toward a goal of addressing all significant variations in life stage (Firestone et. al. 2007; Firestone 2010). The U.S. EPA’s guidance helped inform the development of similar guidance by the World Health Organization (Cohen Hubal et al. 2014).

**Table 1:** Children’s age groups for exposure assessment (U.S. EPA 2005a).

Groups < 1 year old	Groups > 1 year old
Birth to < 1 month	1 to < 2 years
1 to < 3 months	2 to < 3 years
3 to < 6 months	3 to < 6 years
6 to < 12 months	6 to < 11 years
	11 to < 16 years
	16 to 21 years

To support implementation of the guidance, the U.S. EPA published the “Child-Specific Exposure Factors Handbook” (U.S. EPA 2008a). The information in this report has been incorporated in the Agency’s updated document entitled “Exposure Factors Handbook 2011 Edition” (U.S. EPA 2011a). The Handbook provides information on various physiological and behavioral factors to be used in assessing exposure, arrayed by the life stages and age groups as defined in the 2005 guidance on age groupings (U.S. EPA 2005a).

An example of the application of these exposure tools is the use of both the age-grouping guidance and exposure factors handbook data in “Perchlorate Supplemental Request for Comments” (see Table 2 in U.S. EPA 2009). This analysis informed the agency’s 2011 announcement that perchlorate meets the Safe Drinking Water Act criteria for a positive regulatory determination (U.S. EPA 2011b).

***Risk assessment.*** An adage in the field of toxicology attributed to the Swiss scientist Paracelsus (1493–1541) is that “the dose makes the poison.” This simplification does not take into account two potentially important factors. The first involves the issue of timing of exposure or dose, as some chemicals exhibit unique and enhanced toxicity during critical windows of development that can impact the nature and severity of disease (Selevin et al. 2000). The second factor relates to endocrine-disrupting chemicals whose effects may be very different at low doses than at high doses (Vandenberg et al. 2012).

Incorporation of new information regarding early life-stage susceptibility represented a key challenge in the U.S. EPA’s almost 20-year effort to update its 1986 “Guidelines for Carcinogen Risk Assessment” (U.S. EPA 1986). The Agency ultimately developed two guidance documents in 2005—a revised “Guidelines for Carcinogen Risk Assessment” (U.S. EPA 2005b) and a companion “Supplemental Guidance for Assessing Susceptibility from Early-life Exposure to Carcinogens” (U.S. EPA 2005c). The supplemental guidance was not included as a part of the 2005 revised cancer guidelines because the field of early life-stage susceptibility was thought to be actively changing; thus, revising and updating the guidance could be performed in a more expeditious manner than would be required for a major U.S. EPA risk assessment guideline.

The supplemental guidance, based upon an analysis of data for more than 50 chemicals causing cancer through perinatal exposure, concluded that cancer risks generally are higher from early-life exposure than from similar levels of exposure later in life, especially for mutagenic carcinogens. The guidance recommends the use of age-dependent adjustment factors (ADAFs) for those cases where chemical specific data are lacking when assessing cancer risk, but only for carcinogens that act via a mutagenic mode of action:

For exposures before 2 years of age, a 10-fold adjustment.
For exposures between 2 and < 16 years of age, a 3-fold adjustment.
For exposures after turning 16 years of age, no adjustment.

The supplemental guidance states that “development of guidance for estrogenic agents and chemicals acting through other processes resulting in endocrine disruption and subsequent carcinogenesis, for example, might be a reasonable priority in light of the human experience with diethylstilbesterol and the existing early-life animal studies” (U.S. EPA 2005c). Additionally, the National Research Council (NRC) in their “Science and Decisions” report observed that “in practice, EPA treats the prenatal period as devoid of sensitivity to carcinogenicity … and that EPA needs methods for explicitly considering in cancer risk assessment *in utero* exposure and chemicals that do not meet the threshold of evidence that the agency is considering for judging whether a chemical has a mutagenic mode of action. Special attention should be given to hormonally active compounds and genotoxic chemicals that do not meet the threshold of evidence requirements” (NRC 2009).

To follow up on these findings, research is needed in addressing cancer risk resulting from early-life exposure to carcinogens.

## Addressing Children’s Environmental Health through the Development of the U.S. EPA’s Rules and Regulatory Support

In order to implement environmental laws passed by Congress and signed by the President, the U.S. EPA develops and enforces environmental regulations. The Agency has authority under more than twenty statutes, including the Clean Air Act Amendments of 1990 (1990), the Safe Drinking Water Act (2002), and the Toxic Substances Control Act of 1976 (1976). Addressing children’s environmental health in regulatory actions is a central part of the U.S. EPA’s work to protect children. In addition to the three key statutory authorities explicitly calling for children’s health protection—FQPA, Safe Drinking Water Act, and Toxic Substances Control Act—children are generally identified as a sensitive, susceptible, or vulnerable group under the authorities granted by other statutes.

The U.S. EPA’s “Action Development Process: Guide to Considering Children’s Health When Developing EPA Actions” (U.S. EPA 2006b) provides information for considering children’s environmental health in regulatory actions, such as what type of children’s health information should be described, questions for risk assessors, and how to present children’s health considerations to decision makers. Examples in the next section are intended to provide insight into various types of regulatory approaches taken by the U.S. EPA, including updating existing regulatory standards to better protect children, expanding regulatory action that did not previously focus on children, and developing new standards in which protection of children from emerging threats is a key driver.

### National Ambient Air Quality Standards for Lead—2008 Revisions

Since the time of initial publication in 1978, the goal of the National Ambient Air Quality Standards (NAAQS) for lead (Pb) has been to protect children from the neurodevelopmental effects of airborne lead exposure (U.S. EPA 1978). Under authority granted by the Clean Air Act, the 1978 standard set the level at 1.5 μg/m^3^ and acknowledged that young children (1–5 years old) should be regarded as a group within the general population that is particularly sensitive to lead. Based on new scientific information regarding adverse neurodevelopmental outcomes (U.S. EPA 2006a, 2013c), the Agency lowered the Pb NAAQS by an order of magnitude to 0.15 μg/m^3^ in 2008 (U.S. EPA 2008b). The preamble to this revision recognizes that “(t)here is no level of Pb exposure that can yet be identified, with confidence, as clearly not being associated with some risk of deleterious health effects” and further describes children to be at increased risk due to various factors that enhance their exposures such as hand-to-mouth activity.

The public health impact of the U.S. EPA’s regulatory actions, including the banning of lead in paint and automotive gasoline in the 1970s and 1980s, has resulted in a significant reduction of children’s exposure to lead. The U.S. EPA’s “America’s Children and the Environment” (https://www.epa.gov/ace) indicators report shows that the median concentration of lead in the blood of children between the ages of 1 and 5 years dropped from 15 μg/dL in 1976–1980 to 1.0 μg/dL in 2011–2012, a decrease of 93% (U.S. EPA 2015a; see Indicator B1: https://www.epa.gov/ace/biomonitoring-lead).

Several studies have reported that reducing lead exposure was associated with a reduction in criminal behavior (Carpenter and Nevin 2010; Muennig 2009; Wright et al. 2008).

### Restriction in Organophosphate Pesticide Registrations Including Residential Use Cancellations in 2000

Chlorpyrifos and other organophosphate (OP) pesticides are thought to act through a common mode of action by inhibiting nerve function through inhibition of acetylcholinesterase (Mileson et al. 1998). Prior to 2000, chlorpyrifos was the most widely used household pesticide in the United States and was registered for home uses such as outdoor broadcast lawn treatment and indoor crack and crevice treatment. In an effort to protect children, all residential uses (except for roach bait stations in child resistant packaging and fire ant mound treatments) were eliminated in 2000 due to a concern about developmental neurotoxicity of chlorpyrifos (U.S. EPA 2000a).

In addition, many agricultural uses of OPs were also restricted (e.g., tomatoes) or discontinued or cancelled (e.g., apples, citrus, and tree nuts). The risk assessment supporting these actions retained the 10-fold safety factor defined by the FQPA for the protection of children (U.S. EPA 2000b). The U.S. EPA illustrates the reduction in detectable OP pesticide residues in some fruits and vegetables over a 10-year period beginning in the late 1990s (U.S. EPA 2013a; see Indicator E9: https://www.epa.gov/ace/environments-and-contaminants-chemicals-food).

Scientific information on risks associated with chlorpyrifos exposure continues to develop, further illustrating risks to children’s health. Based on a revised risk assessment identifying drinking water and occupational risks (U.S. EPA 2014), the U.S. EPA has proposed a cancellation of all remaining food tolerances for chlorpyrifos (U.S. EPA 2015c).

### Control of Greenhouse Gas Emissions from Light-Duty and Medium- and Heavy-Duty Vehicles—2011–2012

In recent years, the U.S. EPA has established new regulations to address climate change under the authority granted by the Clean Air Act. The final rules controlling emissions of greenhouse gasses from light-duty (cars and light trucks) and medium- and heavy-duty vehicles are examples of such standards (U.S. EPA 2011c, 2012). In limiting greenhouse gas emissions, the U.S. EPA is addressing threats to public health, including children’s health, associated with climate change. In its continued effort to address climate change, the Agency has proposed Phase 2 of the Greenhouse Gas Emissions Standards and Fuel Efficiency Standards for medium- and heavy-duty engines and vehicles (U.S. EPA 2015d), among other actions to adapt and mitigate climate change.

## Children’s Environmental Health Research

Building on the imperative of the Executive Order 13045, the U.S. EPA and the National Institute of Environmental Health Sciences (NIEHS) have jointly funded the Children’s Environmental Health and Disease Prevention Research Centers since 1998. These multidisciplinary research centers examine how environmental factors affect children’s health and promote translation of basic research findings into intervention and prevention methods to prevent adverse health outcomes [see U.S. EPA (https://www.epa.gov/research-grants/niehsepa-childrens-environmental-health-and-disease-prevention-research-centers) and NIEHS (https://www.niehs.nih.gov/research/supported/centers/prevention/)].

Examples of emerging areas of research include understanding the role of environmental factors in the public health epidemic of obesity among our nation’s children; determining how widespread are exposures to chemicals that interfere with the body’s hormones affecting children; and identifying how epigenetic modifications to DNA resulting from diet, aging, stress, and environmental exposures affect our children and our grandchildren.

Recently, the U.S. EPA published its “Children’s Environmental Health Research Roadmap” (U.S. EPA 2015b). In response to the EPA’s unique mandate to understand the role of exposure to environmental hazards during early life, this research roadmap presents a vision for providing integrated, cutting-edge science on children’s environmental health to inform agency decisions. The research described in this roadmap includes efforts by agency researchers as well as externally funded scientists such as the Centers described above.

## Future Challenges

Although the U.S. EPA has taken many actions during the past two decades to improve children’s health protection, important challenges remain. Perhaps the most daunting challenge is understanding and mitigating, to the degree possible, the impacts of climate change on children. The U.S. Global Change Research Program has released a new report—“The Impacts of Climate Change on Human Health in the United States: A Scientific Assessment” (USGCRP 2016) that includes information on those most vulnerable including children and pregnant women. The effects of climate change on children’s health include physical and psychological impacts of weather disasters, increased heat stress, decreased air quality, altered disease patterns, and impacts on the availability of food and clean water (Ahdoot et al. 2015).

Implementing the newly enacted 2016 Frank R. Lautenberg Chemical Safety for the 21st Century Act (2016), which amends the Toxic Substances Control Act, represents an opportunity to improve protection of “potentially exposed or susceptible sub-populations … such as infants, children, (and) pregnant women” from exposure to industrial chemicals. The new provisions allow for greater opportunity to take action to address potential health risks from both new and existing toxic chemicals with explicit considerations for infants, children, and pregnant women.

The U.S. EPA continues to explore incorporating new research in its risk assessment methodology. While many advancements have been made in children’s health risk assessment, there are still many research needs including the following:

Improved understanding of mechanisms leading to early-life windows of susceptibility (e.g., examining possible links between early-life exposure to endocrine disrupters and cancer).
Better characterization of child-relevant exposure pathways such as soil and dust ingestion.
Models that more accurately reflect the dynamic and variable nature of growth that can appropriately represent early-life stages, especially given the expanded use of physiologically based pharmacokinetic models in risk assessment.
Data to illuminate possible links between early-life exposure to environmental chemicals and chronic disease in adulthood (i.e., Barker Hypothesis) (Calkins and Devaskar 2011).
Improved reporting and application of epidemiology findings in risk assessment, so that the observations in children may be better used in decision-making.

There will remain emerging children’s environmental health concerns that have not yet been discovered or addressed. For example, questions about the safety of recycled tire crumb used in athletic fields and playgrounds has recently led to a new federal-wide (including U.S. EPA) effort—Federal Research on Recycled Tire Crumb Used on Playing Fields (https://www.epa.gov/chemical-research/federal-research-recycled-tire-crumb-used-playing-fields). The continued engagement of the research community in children’s environmental health is needed to help address these challenges and protect our nation’s children for generations to come.

## Conclusion

Building on two decades of experience, the U.S. EPA continues to focus its efforts in three principal areas—science to better understand early-life susceptibility, consideration of children in environmental regulations, and outreach to inform health care providers and the public.

Much of the U.S. EPA’s work during the past 20 years to protect children from environmental hazards can be considered primary prevention. These are actions and measures at the population level that minimize hazards to health and that inhibit the emergence and establishment of factors known to increase the risk of disease. For example, the U.S. EPA’s regulations for Pb and OP pesticides have reduced children’s exposures and thus, the potential for neurodevelopmental disease.

The evolution and expansion of children’s environmental health protection over the past two decades has been remarkable. At the U.S. EPA, significant efforts have been made to address the special susceptibility of children, and our work continues to address emerging environmental concerns to ensure that children’s environments are free of hazards and support healthy development.
